# The relationship between smartphone addiction and sleep disorder among college students: negative emotions as a mediator and gender as a moderator

**DOI:** 10.3389/fpsyt.2025.1542243

**Published:** 2025-02-04

**Authors:** Siyi Li, Yingying Deng, Lihong Cai, Linlin Wu

**Affiliations:** ^1^ Higher Education Research Institute, Shantou University, Shantou, China; ^2^ Business School, Xiamen Institute of Technology, Xiamen, China

**Keywords:** college students, smartphone addiction, sleep disorder, negative emotions, gender

## Abstract

**Background:**

College students are disproportionately affected by smartphone addiction, which has been linked to various health impairments, including sleep disorders. This study explores the relationship between smartphone addiction and sleep disorders, with a focus on negative emotions as a mediator and gender as a moderator.

**Methods:**

Cluster sampling was used to survey 1056 Chinese college students. The Mobile Phone Addiction Index, Negative Affect Scale, and Sleep Disorder Scale were administered. Versions of SPSS 27.0 and PROCESS macro 4.0 were used for data analysis, employing Models 15 and 4 to examine moderation and mediation, respectively.

**Results:**

A strong direct correlation was identified between smartphone addiction and sleep disorder, with negative emotions mediating this relationship, accounting for 33.7% of the effect. Gender significantly moderated the mediation process, with females demonstrating a greater correlation than males between sleep disorders and negative emotions.

**Conclusion:**

Negative emotions partially mediated smartphone addiction’s association with sleep disorder, whereas gender significantly moderated this mediation. These findings underscore the complex dynamics among smartphone addiction, emotional well-being, and sleep among college students.

## 1 Introduction

Smartphones play a significant role in modern life, particularly in social interaction, entertainment, and learning, which has led to an increasing reliance on them, especially among adolescents. For instance, among Spanish children aged 10-15, approximately 70% own a smartphone, and ownership rises with age; among teenagers 16 and older, it reaches 99.1% (1). According to the latest 2024 survey on the scale of internet users in China, the number of Chinese internet users grew by 7.42 million compared to 2023, with adolescents representing the largest group, accounting for 49% of the new internet users (2). Despite the convenience that smartphones offer in daily life, research has shown that frequent use and dependence on smartphones can lead to addictive behavioral patterns (3, 4). Smartphone addiction is defined as the use of smartphones to fulfill deep-seated needs (such as dependence, habitual use, and addictive behaviors), to the extent that individuals find it impossible to accomplish essential daily chores without utilizing their smartphones (5). Studies indicate that 31.6% of adolescents in South Korea are addicted to smartphones (6), and Over 25% of Chinese teenagers suffer from smartphone addiction (7). Smartphone addiction can severely impact adolescents’ physical health (8), academic performance (5), emotional well-being (9), and sleep quality (10).

Studies have indicated that negative emotions such as loneliness and anxiety are predictive factors for college students’ addiction to smartphoness (11). The cognitive-behavioral hypothesis states that behaviors can also reciprocally influence emotions and cognition (12). Therefore, individuals exhibiting addictive smartphone behaviors may struggle to control excessive use, which may result in negative emotions such as worry, anxiety (13), and depression (14). These negative emotions, in turn, can further affect sleep quality (15). College students often engage in frequent internet use (16) and tend to have weaker self-regulation skills (17), making them more susceptible to smartphone addiction. Poor sleep quality is significantly influenced by smartphone addiction (18, 19), and existing research has confirmed a direct association between sleep disorders and smartphone addiction among adolescents (20). For instance, a bidirectional relationship exists between smartphone addiction and sleep disorders (21, 22). Some studies also indicate that smartphone addiction and sleep quality are significantly positively correlated (23). Specifically, young individuals with smartphone addiction are are more likely to develop sleep disorders (20).

In summary, previous studies have provided valuable insights into smartphone addiction’s association with sleep disorders. But little is known about the mediating and regulating mechanisms that underlie this association. The purpose of this study is to investigate whether the mediating role of negative emotions and the moderating role of gender can reduce the impact of smartphone addiction on sleep disorders, offering valuable insights for the prevention and management of sleep disorders caused by smartphone addiction among college students.

### 1.1 The mediating role of negative emotions

Emotions are a combination of brain and body responses that manifest as our feelings and outward appearances (24). In general, people can easily distinguish between positive and negative emotions (25). Negative emotion is a relatively low emotional response (26), including depression (27), anger, frustration and other negative psychological effects (28). In recent years, there have been a number of studies linking it to smartphone addiction (29, 30). In the beginning, smartphone use can be enjoyable and exciting for individuals (31). However, when the phenomenon of smartphone addiction occurs, it is often impossible to control excessive dependence on smartphones, which leads to negative emotions being activated (32), making it seem anxious (33) and depressed (34, 35). At the same time, smartphone addiction may exacerbate an individual’s fear of missing out on information (36), and may even lead to depression, anger, and, in severe cases, an increased risk of suicide (37).

Many factors can influence sleep, including nutrition, use of stimulants (alcohol, caffeine, and tobacco), physical activity levels (38), shoulder pain and disability (39), physical environment, and social environment (40). In addition, emotional state is also an important factor affecting sleep, and different emotional experiences may even lead to the occurrence of sleep disorders (41). Sleep disorders are physiological problems resulting from disturbances in the regulation mechanisms of the sleep and wake cycles, and are often characterized by symptoms such as insomnia, sleep deprivation, poor sleep quality, and impaired daytime functioning (20). Negative emotions are strongly associated with sleep disorders (42). For example, research has shown that brain structures associated with negative emotions are significantly correlated with sleep disorders in normal young people (43). Previous studies have also confirmed the influence of negative emotions such as anxiety (44), depression (45) and stress on sleep disorders (46). For example, studies have found that college students with depressive symptoms are more likely to have sleep disorders (47). In addition, evidence suggests that college students with high levels of negative emotions are at a greater risk of experiencing sleep disorders (36).

Research has revealed a big positive association between smartphone addiction and sleep disorders. Psychological resilience and physical exercise behavior play a partial mediating role in this relationship (10, 48). Therefore, the effect of smartphone addiction on sleep disorders may be indirectly through mediating variables rather than directly. This finding suggests that further exploration of the mediating effect of smartphone addiction on sleep disorders in college students is needed, with additional empirical studies required to uncover the underlying mechanisms.

### 1.2 The moderating role of gender

The concept of “gender” is deconstructed into four aspects: biological/physical traits, self-defined gender identity, legal gender, and gender expression (49). The term gender is a straightforward description of some of the most basic human characteristics, but when individuals are asked their gender they are usually given two choices: male or female (50). The gender differences show up in different individual variables. Specifically, a number of studies have observed that smartphone addiction varies by gender, such as studies that categorize smartphone users into three potential categories, while women are placed in the high-risk category where smartphone addiction is more prevalent among women than men (51). A study of Chinese college students found that female undergraduates were more likely than male undergraduates to use smartphones excessively (52). Gender differences also show up in negative emotions. The study found that female undergraduates’ average anxiety levels were noticeably greater than those of male students (53). Additionally, research indicates that female undergraduates experience higher levels of stress than their male counterparts (54). These results suggest that female undergraduates may face more negative emotional challenges. This gender difference is also reflected in sleep problems, which show up as early as infancy and continue into childhood and adulthood (55), and are particularly pronounced in young adults (56). Specifically, female college students are less sleep efficient than male college students (57). Moreover, female undergraduates are more likely to experience sleep disorders than male undergraduates (58).

Based on the above research, the role of gender in the three variables of smartphone addiction, negative emotions and sleep disorders should not be ignored. Although previous research has revealed significant differences between gender in individual variables, there is still insufficient research on how gender regulates the relationship between these variables. With this in mind, this study aimed to explore whether gender is a moderating factor that influences the direct and/or indirect association between smartphone addiction and sleep disorders. By constructing a theoretical model that incorporates gender-mediated effects, this study will provide a new perspective for understanding the role of gender in the path of smartphone addiction-induced sleep disorders and may reveal gender-specific prevention and intervention strategies.

Hypothesis 2: Smartphone addiction’s association with sleep disorders, both directly and indirectly, varies by gender, with negative emotions acting as a mediating factor.

**Figure 1:**
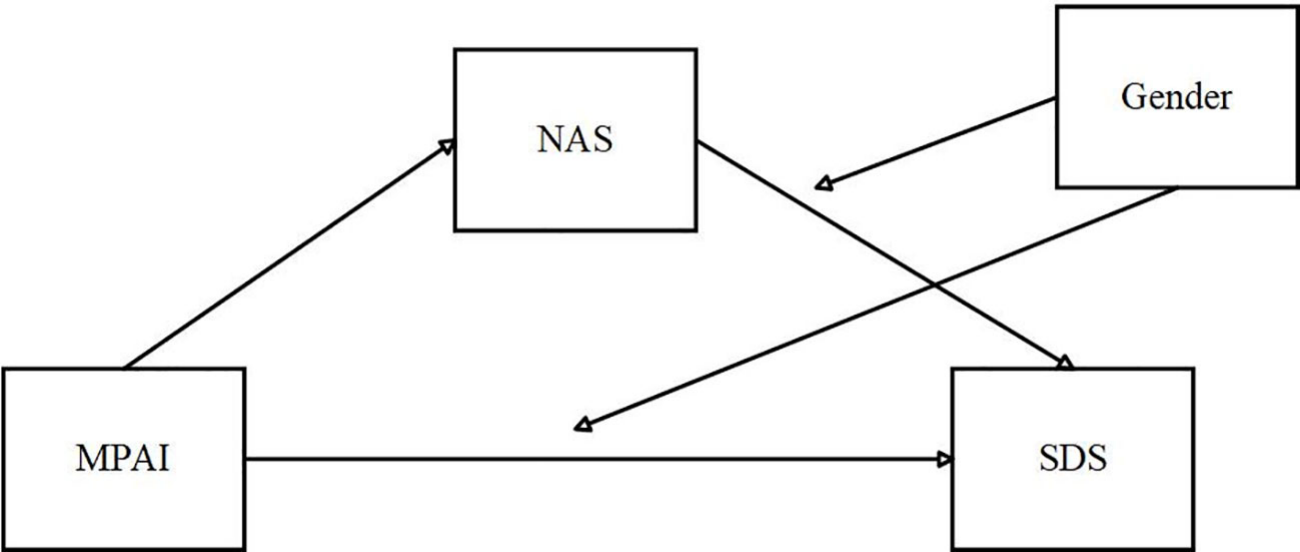
Model of the hypotheses in this research.

**Figure 1** displays the study’s hypothesized model.

## 2 Methodology

### 2.1 Participants and processes

Using cluster sampling, an online questionnaire was administered from October 8 to October 28, 2024, to measure smartphone addiction, negative emotions, and sleep disorders among students pursuing various college degrees across 20 provinces and cities. Prior to the survey, teachers provided guidance through class WeChat groups, obtained informed consent from participants, and introduced the purpose, use, and significance of the data collected while emphasizing test anonymity, confidentiality, and voluntary participation. Participants were given full autonomy to discontinue or opt out of the questionnaire at any time. This study was approved by the Ethics Committee for Scientific and Technological Research Involving Human Beings of Shantou University (approval number: STU202410004).

Overall, 1056 questionnaires were gathered. Questionnaires were ruled invalid if they omitted answers, selected the same option consecutively more than 10 times, or had response times of less than 2 minutes. Based on these screening criteria, 123 samples were excluded, resulting in 933 valid responses, equivalent to an 88.4% validity rate. Additionally, general personal information such as gender, place of origin, grade level, only-child status, and family structure (e.g., belonging to a two-parent family) was gathered.

### 2.2 Measures

#### 2.2.1 Mobile phone addiction index

In this study, smartphone addiction among college students was evaluated using MPAI, created by Liang Yongchi (59). The scale comprises 17 items spanning four dimensions: uncontrollability, escapism, withdrawal symptoms, and inefficiency. Each item is rated on a 5-point Likert scale, where 1 denoting “never” and 5 indicating “always” (60). Furthermore, a higher overall score indicates a higher degree of smartphone addiction. The MPAI’s Cronbach’s alpha was 0.93. Besides, the Kaiser-Meyer-Olkin value was 0.941.

#### 2.2.2 Negative affect scale

This study employed the NAS from Watson et al.’s comprehensive emotion assessment tool (61). The NAS evaluates the intensity of negative emotions experienced over a specific timeframe, encompassing a range of unpleasant emotional states such as anger, guilt and tension. Ten items on a Likert scale of five points make up NAS, and the overall range is from 10 to 50. Greater negative emotion intensity is indicated by a higher score. It was discovered that the NAS’s Cronbach’s alpha was 0.89. Besides, the Kaiser-Meyer-Olkin value was 0.900.

#### 2.2.3 Sleep disturbance scale

To assess participants ‘ sleep disorder levels, the study utilized the SDS, a component of the Pittsburgh Sleep Quality Index (PSQI) developed by Daniel et al. (62). This scale assesses the frequency of 9 specific sleep-related issues, including “difficulty staying asleep or waking up early,” “breathing difficulties,” “coughing or loud snoring,” and “having nightmares.” Each response is assessed using a scale made of four points between 0 and 3, corresponding the following categories: “none,” “less than once per week,” “1-2 times per week,” and “more than three times weekly,” respectively. The SDS demonstrated a Cronbach’s alpha of 0.72, reflecting reasonable reliability. Besides, the Kaiser-Meyer-Olkin value was 0.827.

### 2.3 Data processing and analysis

Valid data were entered in SPSS 27.0 for analysis, with all statistical operations carried out using this software. First, Kolmogorov-Smirnov (K-S) non-parametric test alongside reliability analysis were utilized to evaluate the normality of data distribution and the reliability of the instruments. Next, after standardizing the data, descriptive statistics were conducted for demographic variables, and differences in research variables across gender were examined using the Mann-Whitney U test. Subsequently, Hayes ‘ PROCESS macro (Model 4) (63) was used to examine the mediating role of negative emotions in the relationship between smartphone addiction and sleep disorders. To facilitate the analysis, the independent variable, mediator, and moderator were mean-centered. Finally, Hayes’ PROCESS macro (Model 15) (64) was used to examine the moderating effect of gender within the mediation model.

## 3 Results

### 3.1 Common method bias test

For evaluating potential biases from the common method, the Harman single-factor test was performed. Excluding demographic variables, an analysis of the unrotated exploratory factors was conducted for the remaining items. Six factors that had eigenvalues above 1 were identified, with the first factor explaining 27.767% of the variance—substantially less than the 40% threshold. Therefore, this study did not have significant bias in the common method.

### 3.2 Descriptive analysis

The demographic variables related to the study, including gender, place of origin, only-child status, family structure (e.g., belonging two-parent family), and grade level, are detailed in **Table 1**.

**Table 1:** Summary of overall sample characteristics.

Variables	Number	Percentage
Gender	933	100%
Male	337	36.10%
Female	596	63.90%
Residence	933	100%
Rural	496	53.20%
Urban	437	46.80%
Only child or not	933	100%
Yes	331	35.50%
No	602	64.50%
Type of family	933	100%
Two-parent family	805	86.30%
Else	128	13.70%
Academic years	933	100%
Freshman	531	56.90%
Sophomore	144	15.40%
Junior	178	19.10%
Senior	80	8.60%

For the sample population, MPAI’s mean was 28.76 ± 12.22, NAS’s mean was 22.99 ± 7.53, and SDS’s mean was 4.53 ± 4.26. Furthermore, our study examined gender differences across research variables, as indicated by **Table 2**. The mean MPAI score among males was significantly higher than that among females, with males scoring significantly higher in specific dimensions, including uncontrollability, escapism, withdrawal symptoms, and inefficiency. Conversely, females scored considerably higher than males on the SDS.

**Table 2:** Gender differences in variables: results of the Mann-Whitney U test.

Variables	Full example	Male	Female	P
	$\bar{x}$ ±s	$\bar{x}$ ±s	$\bar{x}$ ±s	
MPAI				
Total	28.76±12.22	33.52±13.14	26.07±10.79	0.000***
Uncontrollability	11.51±5.18	13.54±5.76	10.37±4.43	0.000***
Escapism	6.19±3.02	7.25±3.39	5.59±2.60	0.000***
Withdrawal Symptoms	5.75±2.93	6.67±3.14	5.23±2.67	0.000***
Inefficiency	5.30±2.72	6.05±2.86	4.88±2.55	0.000***
NAS	22.99±7.53	23.61±7.90	22.65±7.30	0.077
SDS	4.53±4.26	4.09±3.92	4.78±4.42	0.008**

**P<0.01, ***P<0.001.

### 3.3 Correlation analysis

To control for demographic variables, a partial correlation analysis was conducted after controlling for gender. The findings, presented in **Table 3**, reveal significant positive association between smartphone addiction and negative emotions (r = 0.342, p < 0.001) and between smartphone addiction and sleep disorders (r = 0.259, p < 0.001). Additionally, negative emotions were significantly directly associated with sleep disorders. (r = 0.314, p < 0.001).

**Table 3:** Partial correlation analysis of variables.

Control Variable	Variable	Negative emotion	Sleep disorder	Smartphone addiction
Gender	Negative emotion	1		
	Sleep disorder	0.314***	1	
	Smartphone addiction	0.342***	0.259***	1

***P<0.001.

### 3.4 Analysis of the effect of mediation

The mediating role of negative emotions in the relationship between smartphone addiction and sleep disorders was examined using Hayes ‘ (2012) SPSS macro Model 4, with gender controlled for. As shown in **Table 4**, smartphone addiction showed a significant positive correlation with negative emotions, and both were significantly positively associated with sleep disorders.

**Table 4:** Examining negative emotions ’ mediation regarding smartphone addiction’s association with sleep disorder among college students.

	Negative emotion				Sleep disorder			
			Bootstrap 5000 Times 95%CI				Bootstrap 5000 Times 95%CI	
	β	S.E.	LLCI	ULCI	β	S.E.	LLCI	ULCI
Smartphone addiction	0.357	0.032	0.294	0.420	0.179	0.034	0.113	0.246
Negative emotion	-	-	-	-	0.255	0.033	0.191	0.319
Gender	0.091	0.067	-0.041	0.222	0.303	0.067	0.173	0.434

**Figure 2:**
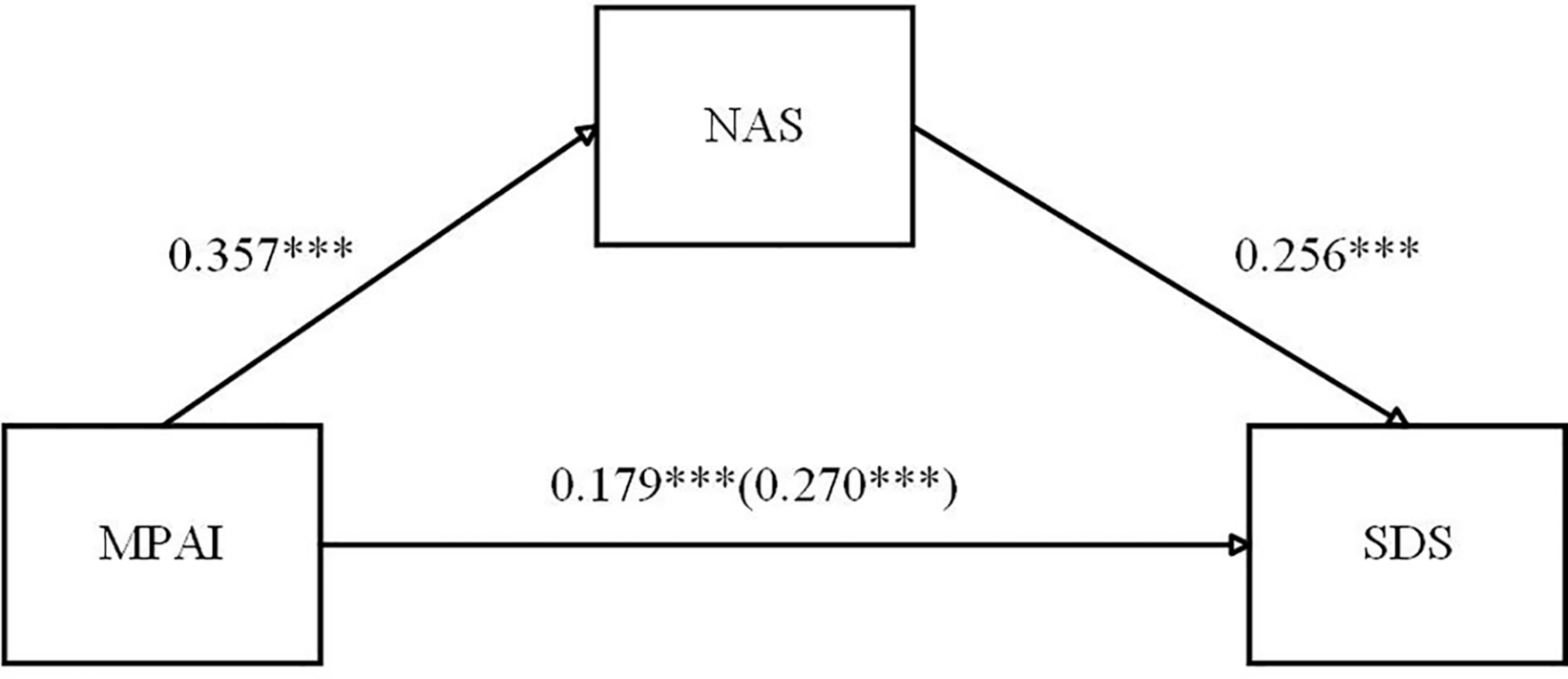
Effect of MPAI on SDS with the mediation of NAS. ***P < 0.001.

**Figure 2** presents the mediation model, and the findings reveal that all three pathways were statistically significant. The results indicated that smartphone addiction’s overall influence against sleep disorders was 0.270 (p < 0.001). The direct influence was 0.179, with a 95% CI of 0.113–0.246, representing 66.3% of the total influence. The indirect effect mediated by negative emotions was 0.091, with a 95% CI of 0.055–0.132, accounting for 33.7% of the overall influence. These findings suggest that negative emotions mediate smartphone addiction’s association with sleep disorders only partially, supporting Hypothesis 1.

### 3.5 Analysis of the effect of moderated mediation

In Hypothesis 2, we hypothesize that gender moderates the direct and indirect effects of smartphone addiction on sleep disorders in a mediation model involving smartphone addiction, negative emotions, and sleep disorders. To test this hypothesis, the study used Hayes’ PROCESS macro (Model 15) to investigate the moderating effect of gender in the mediation model. Specifically, Hypothesis 2 includes two specific sub-hypotheses: (a) gender moderates smartphone addiction’s association with sleep disorders, and (b) gender moderates negative emotions’ association with sleep disorders. If Hypothesis b is supported, it indicates the existence of a moderated mediation model.

The outcomes that **Table 5** presented indicate that gender did not moderate the direct path but influenced the latter portion of the mediation pathway. Specifically, the interaction term between smartphone addiction and gender was insignificant predictor of sleep disorders (β = 0.009, p > 0.05). However, the interaction between gender and negative emotions significantly predicted sleep disorders (β = 0.113, p < 0.01), suggesting gender moderates the link between negative emotions and sleep disorders, thus supporting Hypothesis b. Therefore, the results suggest that Hypothesis 2 is partially supported, and a moderated mediation model is established.

**Table 5:** Effect of moderated mediation between addiction to smartphone and sleep disorder.

Predictor	NAS		SDS	
	β	t	β	t
MPAI	0.212	11.195***	0.053	1.328
NAS	–	–	-0.039	-0.602
Gender	–	–	1.284	4.510***
MPAI*Gender	–	–	0.009	0.371
NAS*Gender	–	–	0.113	2.960**
R2	0.119		0.140	
F	125.334***		30.256***	
Conditional indirect influence with respect to gender				
Gender	Effect	BootSE	Bootstrap 5000 Times 95%CI	
			LLCI	ULCI
Male	0.016	0.007	0.003	0.030
Female	0.040	0.009	0.024	0.058
Moderated mediation index	0.024	0.010	0.005	0.045

**P<0.01, ***P<0.001.

**Figure 3:**
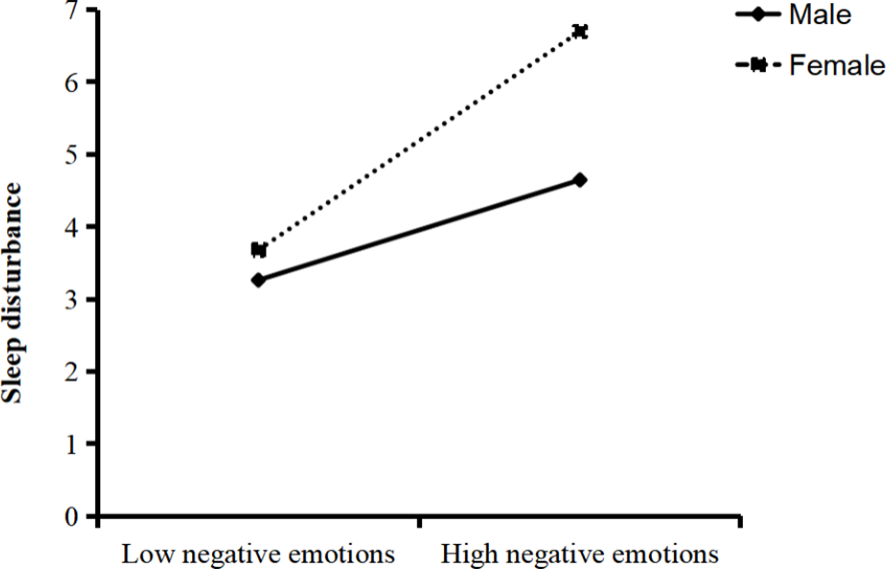
Gender’s moderation in negative emotions and sleep disorder.

To specifically illustrate how gender moderates the connection between negative emotions and sleep disorders, the study plotted simple slope graphs for negative emotions and sleep disorders, as shown in **Figure 3**. In the female group, negative emotions significantly predicted sleep disorders (simple slope = 0.187, t = 8.113, p < 0.001); whereas in the male group, the association between negative emotions and sleep disorders was weaker (simple slope = 0.074, t = 2.422, p = 0.016). This suggests that the female group experiences more sleep disorders as negative emotions increase.

## 4 Discussion

Sleep disorder and smartphone addiction have been found to be strongly correlated (65–67). Numerous studies have demonstrated that smartphone addiction contributes to sleep disorder (68).

However, the mediating and regulating mechanisms that account for the connection between smartphone addiction and sleep disorders have not been thoroughly studied. To address these gaps, this study enhances the current literature via constructing a model for moderated mediation. This model investigates how negative emotions mediate smartphone addiction’s association with sleep disorders in college students and explores the moderating role of gender on this mediation. These findings suggest that first association is mediated by negative emotions. Specifically, negative emotions positively predict sleep disorders, whereas smartphone addiction predicts negative emotions. In addition, negative emotions’ association with disturbance during sleep was moderated by gender. This topic is explored in the subsequent section based on the study’s findings.

### 4.1 Negative emotions’ mediation

Consistent with previous researches (20, 21, 69), we found that addiction to smartphone among college students directly associates with sleep disorders. In other words, greater addiction to smartphones was associated with greater sleep disorder. Studies have revealed that college students frequently utilize smartphones for text messaging, phone calls, and gaming (70). This suggests that as the amount of time college students spend on their phones increases, their sleep time decreases accordingly. According to the sleep disorder process theory, heightened emotional or physiological arousal negatively affects sleep quality (71). For instance, the initiation of sleep and the occurrence of mood swings or awakenings during sleep significantly affect sleep quality. Pre-sleep smartphone usage and the type of content browsed contribute to psychological stress and physiological arousal (72), ultimately compromising sleep quality. Furthermore, electromagnetic fields emitted by smartphones can disrupt sleep structure (73), and the use of light-emitting electronic devices suppresses melatonin, extending the time required to fall asleep (74). Collectively, these factors can lead to sleep disorders in college students.

Furthermore, our findings indicate that negative emotions partially mediate the connection between smartphone addiction and sleep disorders. A positive relationship was observed between negative emotions and addiction to smartphone, where higher addiction levels corresponded to increased negative emotions. These findings align with those of Chen et al. (75), who argued that individuals addicted to or at risk of addiction experience greater negative emotions due to the financial and time investments involved. Moreover, some studies have explored how smartphone addiction contributes to negative emotions. For example, Çağan et al. (76) found that the degree of addiction to smartphone is directly associated with depression. Similarly, Demirci et al. (66) identified a direct relationship between smartphone addiction and both depression and anxiety. Jun (14) further demonstrated that smartphone addiction affects depressive symptoms over time. Additionally, we observed a positive relationship between negative emotions and sleep disorders, consistent with previous studies. For instance, research has shown that negative emotions significantly and positively predict sleep quality (23). According to the “conflict internalization model” of insomnia, internalizing psychological conflict increases emotional arousal and physiological activation, negatively affecting sleep (77). Furthermore, studies have demonstrated that negative emotions, such as worry and stress can result in sleep disorders. Jansson-Fröjmark and Lindblom (78) found that high levels of anxiety and depression predict insomnia. Similarly, Staner (79) observed that sleep disorders (especially insomnia) are prevalent in individuals with anxiety disorders. Vandekerckhove et al. (80) reported that stress and emotional factors heighten vulnerability to sleep disorders. Integrating these findings, we used a mediation model method to examine the link between negative emotions and sleep disorder and between smartphone addiction and negative emotions. Our study revealed that college students with greater smartphone addiction experienced more negative emotions, increasing their likelihood of sleep disorders.

These findings offer valuable insights into how negative emotions influence sleep disorders in the context of smartphone addiction and lay the groundwork for strategies aimed at addressing and improving the physical and mental health challenges faced by college students. The findings of this study highlight the critical need to address the negative emotions associated with smartphone addiction among college students. Resource conservation theory suggests that individual actively increase resources to mitigate stress rather than passively waiting for a stressful environment to occur (81). However, smartphone addiction often leads to unpleasant emotions such as worry, stress, and despair. Cognitive emotion regulation strategies are vital for addressing negative events and anxiety and depressive symptoms. Effective regulation can reduce negative emotions and the resource consumption caused by smartphone addiction (82). For example, Liang et al. (83) showed that cognitive emotion regulation affects emotional experience and reduced smartphone addiction and its associated negative emotions. Therefore, parents and teachers should consciously guide college students to learn cognitive emotion regulation strategies, improving emotion regulation skills (65) to reduce negative emotions caused by smartphone addiction and enhance sleep quality. Parents can actively participate in “emotional socialization behavior” in family education, actively guide children’s emotional behavior, and construct a good emotional regulation process (84).Teachers can teach strategies related to cognitive emotion regulation, such as mindfulness techniques in the form of meditation, deep breathing, and focus on the present moment (85), cognitive reassessment (helping individuals re-evaluate maladaptive situations and enhance emotional self-control), and expressive inhibition (inhibiting emotional expression behaviors) (83). In addition, parents and teachers can guide college students to relieve their negative emotional experiences through group impromptu music therapy (a way to release emotions and express themselves through group improvisation of Musical Instruments) and tai chi exercises (86, 87), thereby reducing the occurrence of sleep disorders. To enhance sleep quality among college students, interventions targeting smartphone addiction and emotional regulation are essential.

### 4.2 Gender’s moderating effect

Further findings confirmed that gender moderates the indirect influence of smartphone addiction on sleep disorders via negative emotions. Specifically, while negative emotions among college students were significantly correlated with sleep disorders, the effect was stronger in females. Our findings align with prior research suggesting that females respond more strongly to negative stimuli than males (88, 89). For example, all sleep difficulties in females were significantly related to stress, emotional regulation, and negative emotions, whereas in males, only difficulties initiating sleep were associated with these factors (90). Consequently, women experience a broader range of sleep disorders when facing negative emotions. This difference may be linked to gender differences in emotion regulation. Two categories of emotion regulation are distinguished under the process model of emotion regulation: one of which is cognitive reappraisal, a strategy used to reinterpret a situation and mitigate the impact of negative emotions (91). Research has shown that poorer sleep quality is associated with a reduced ability to regulate negative emotions through cognitive reappraisal (92). Structural differences in the brains of males and females contribute to differences in their ability to use cognitive reappraisal. McRae et al. (90) argue that males exhibit lower amygdala activity and less prefrontal cortex activity in emotional responses, with the prefrontal cortex being typically involved in cognitive and emotional control. As a result, males may be more likely than females to spontaneously employ cognitive reappraisal strategies, thereby reducing the negative impact of negative emotions on sleep quality.

Based on these findings, schools and society should focus on addressing sleep disorders among college students and establish corresponding support systems to provide help to college students, especially female students. Schools can provide emotion regulation training courses to help students identify and adjust their emotions using strategies such as cognitive reassessment, mindful acceptance, and other such strategies to mitigate the detrimental effects of negative emotions on individuals (93).Society can also provide college students with psychological counseling services. Given that women are more prone to negative emotions throughout their lives (94), targeted support should account for their psychological and physiological characteristics.

### 4.3 Limitations

Although the participants in this study were college students and the results indicated that negative emotions significantly influenced sleep disorders in female students, these findings may also apply to other groups, such as professionals and adolescents, who might experience similar emotional distress and sleep-related issues. Therefore, future research should explore how these relationships manifest across different populations to enhance the generalizability of these findings.

In addition, several limitations of this study should be considered. First, the use of online questionnaires may have influenced the accuracy of the survey findings as the results are more likely to reflect the views of individuals willing to complete the survey. This limitation could affect the generalizability of the research outcomes. Second, reliance on participants ‘ subjective assessment without objective data verification may have introduced inaccuracies. Future studies could address this limitation by incorporating objective assessment methods such as actigraphy and sleep-tracking applications to enhance the reliability of sleep evaluations. Third, this study measured sleep disorders through self-reports rather than clinical diagnoses, making the findings less applicable to clinical populations. Finally, it is impossible to establish causal links between the variables in this study due to its cross-sectional nature. The causal relationships between smartphone addiction, negative emotions, and sleep disorders should be further examined in future longitudinal investigations. Notwithstanding these drawbacks, the study’s conclusions are nonetheless important. These results highlight the significant predictive role of smartphone addiction in sleep disorders and demonstrate, through mediation analysis, that negative emotions exacerbate the effect of smartphone addiction on sleep disorders. In addition, gender as an individual characteristic also moderates addiction to smartphone in relation to sleep disorders.

### 4.4 Conclusion

This research seeks to unveil how negative emotions mediate smartphone addiction’s association with sleep disorders with respect to college students, as well as moderating influence of gender. It elucidates the interconnected pathways between phone addiction and sleep disorders by examining mediators and moderators. For college students, negative emotions were identified as key mediators of smartphone addiction’s relationship with sleep disorders. Additionally, gender moderated this mediation process, with female students exhibiting a stronger effect of negative emotions on sleep disorders. These findings emphasize the importance of the mediating model in understanding how smartphone addiction contributes to sleep disorder among college students.

## Data Availability

The original contributions presented in the study are included in the article/supplementary material. Further inquiries can be directed to the corresponding author.

## References

[1] Ricoy MC Martínez-Carrera S. Digital newspapers’ perspectives about adolescents’ smartphone use. Sustainability. (2021) 13:5316. doi: 10.3390/su13095316

[2] China Internet Network Information Center. China statistical report on internet development(2024). Available online at: https://www.cnnic.net.cn/n4/2024/0829/c88-11065.html (Accessed November 5, 2024).

[3] Griffiths M. A ‘components’ model of addiction within a biopsychosocial framework. J Subst Use. (2005) 10:191–7. doi: 10.1080/14659890500114359

[4] Lee H Ahn H Choi S Choi W. The SAMS: Smartphone addiction management system and verification. J Med Systems. (2014) 38:1–10. doi: 10.1007/s10916-013-0001-1 24395031

[5] Sunday OJ Adesope OO Maarhuis PL. The effects of smartphone addiction on learning: a meta-analysis. Comput Hum Behav Rep. (2021) 4:100114. doi: 10.1016/j.chbr.2021.100114

[6] Lee C Lee SJ. Prevalence and predictors of smartphone addiction proneness among Korean adolescents. Children Youth Serv Review. (2017) 77:10–7. doi: 10.1016/j.childyouth.2017.04.002

[7] Lu G Ding Y Zhang Y Huang H Liang Y Chen C. The correlation between mobile phone addiction and coping style among Chinese adolescents: a meta-analysis. Child Adolesc Psychiatry Ment Health. (2021) 15:1–11. doi: 10.1186/s13034-021-00413-2 34654451 PMC8520246

[8] Wang J Hao Q Peng W Tu Y Zhang L Zhu T. Relationship between smartphone addiction and eating disorders and lifestyle among Chinese college students. Front Public Health. (2023) 11:1111477. doi: 10.3389/fpubh.2023.1111477 37275494 PMC10235600

[9] Volungis AM Kalpidou M Popores C Joyce M. Smartphone addiction and its relationship with indices of social-emotional distress and personality. Int J Ment Health Addiction. (2020) 18:1209–25. doi: 10.1007/s11469-019-00119-9

[10] Zhu W Liu J Lou H Mu F Li B. Influence of smartphone addiction on sleep quality of college students: the regulatory effect of physical exercise behavior. PloS One. (2024) 19:e0307162. doi: 10.1371/journal.pone.0307162 39058670 PMC11280214

[11] Enez Darcin A Kose S Noyan CO Nurmedov S Yilmaz O Dilbaz N. Smartphone addiction and its relationship with social anxiety and loneliness. Behav Inf Technology. (2016) 35:520–5. doi: 10.1080/0144929X.2016.1158319

[12] Xu L Wu L Geng X Wang Z Guo X Song K. A review of psychological interventions for internet addiction. Psychiatry Res. (2021) 302:114016. doi: 10.1016/j.psychres.2021.114016 34087672

[13] De-Sola Gutiérrez J Rodríguez de Fonseca F Rubio G. Cell-phone addiction: a review. Front Psychiatry. (2016) 7:175. doi: 10.3389/fpsyt.2016.00175 27822187 PMC5076301

[14] Jun S. The reciprocal longitudinal relationships between mobile phone addiction and depressive symptoms among Korean adolescents. Comput Hum Behavior. (2016) 58:179–86. doi: 10.1016/j.chb.2015.12.061

[15] Wang S Matsuda E. The effects of stressful life events and negative emotions in relation to the quality of sleep: a comparison between Chinese and Japanese undergraduate students and Chinese international students. Japanese psychol Res. (2023) 65:99–111. doi: 10.1111/jpr.12363

[16] Li L Xu DD Chai JX Wang D Li L Zhang L. Prevalence of internet addiction disorder in Chinese university students: a comprehensive meta-analysis of observational studies. J Behav Addictions. (2018) 7:610–23. doi: 10.1556/2006.7.2018.53 PMC6426360 30010411

[17] Li Y Li G Liu L Wu H. Correlations between mobile phone addiction and anxiety, depression, impulsivity, and poor sleep quality among college students: a systematic review and meta-analysis. J Behav Addictions. (2020) 9:551–71. doi: 10.1556/2006.2020.00057 PMC8943681 32903205

[18] Sohn SY Krasnoff L Rees P Kalk NJ Carter B. The association between smartphone addiction and sleep: a UK cross-sectional study of young adults. Front Psychiatry. (2021) 12:629407. doi: 10.3389/fpsyt.2021.629407 33737890 PMC7961071

[19] Lane HY Chang CJ Huang CL Chang YH. An investigation into smartphone addiction with personality and sleep quality among university students. Int J Environ Res Public Health. (2021) 18:7588. doi: 10.3390/ijerph18147588 34300037 PMC8307286

[20] Zhang J Zhang X Zhang K Lu X Yuan G Yang H. An updated of meta-analysis on the relationship between mobile phone addiction and sleep disorder. J Affect Disord. (2022) 305:94–101. doi: 10.1016/j.jad.2022.02.008 35149136

[21] Kang Y Liu S Yang L Xu B Lin L Xie L. Testing the bidirectional associations of mobile phone addiction behaviors with mental distress, sleep disturbances, and sleep patterns: a one-year prospective study among Chinese college students. Front Psychiatry. (2020) 11:634. doi: 10.3389/fpsyt.2020.00634 32765310 PMC7379372

[22] Chen JK Wu WC. Reciprocal relationships between sleep problems and problematic smartphone use in Taiwan: cross-lagged panel study. Int J Environ Res Public Health. (2021) 18:7438. doi: 10.3390/ijerph18147438 34299887 PMC8306125

[23] Tao Y Liu Z Huang L Liu H Tian H Wu J. The impact of smartphone dependence on college students’sleep quality: the chain-mediated role of negative emotions and health-promoting behaviors. Front Public Health. (2024) 12:1454217. doi: 10.3389/fpubh.2024.1454217 39363983 PMC11446859

[24] Frenzel AC Daniels L Burić I. Teacher emotions in the classroom and their implications for students. Educ Psychol. (2021) 56:250–64. doi: 10.1080/00461520.2021.1985501

[25] Wang Y Shangguan C Gu C Hu B. Individual differences in negative emotion differentiation predict resting-state spontaneous emotional regulatory processes. Front Psychol. (2020) 11:576119. doi: 10.3389/fpsyg.2020.576119 33244304 PMC7684205

[26] Xiong SC Yuan MQ Zhang B Li YX. College students’ loneliness and mobile phone addiction: The mediating role of negative emotions and negative coping styles. Chin J Health Psychol. (2018) 26:1857–61. doi: 10.13342/j.cnki.cjhp.2018.12.029

[27] Hou J Zhu Y Fang X. Mobile phone addiction and depression: multiple mediating effects of social anxiety and attentional bias to negative emotional information. Acta Psychologica Sinica. (2021) 53:362–73. doi: 10.3724/SP.J.1041.2021.00362

[28] Feng JP Yan X Lu YL Xu JF Sun B Feng LS. Advances in exercise detoxification research. Chin Sports Technol. (2019) 55:3–11. doi: 10.16470/j.csst.2019626

[29] Shen G Huang G Wang M Jian W Pan H Dai Z. The longitudinal relationships between problematic mobile phone use symptoms and negative emotions: a cross-lagged panel network analysis. Compr Psychiatry. (2024) 135:152530. doi: 10.1016/j.comppsych.2024.152530 39303373

[30] Elhai JD Gallinari EF Rozgonjuk D Yang H. Depression, anxiety and fear of missing out as correlates of social, non-social and problematic smartphone use. Addictive behaviors. (2020) 105:106335. doi: 10.1016/j.addbeh.2020.106335 32062337

[31] Li L Griffiths MD Mei S Niu Z. Fear of missing out and smartphone addiction mediates the relationship between positive and negative affect and sleep quality among Chinese university students. Front Psychiatry. (2020) 11:877. doi: 10.3389/fpsyt.2020.00877 33192635 PMC7481466

[32] Seki T Hamazaki K Natori T Inadera H. Relationship between internet addiction and depression among Japanese university students. J Affect Disord. (2019) 256:668–72. doi: 10.1016/j.jad.2019.06.055 31299448

[33] Gong L Liu Q. Mobile phone addiction and sleep quality: the mediating role of anxiety and the moderating role of emotion regulation. Behav Sci. (2023) 13:250. doi: 10.3390/bs13030250 36975275 PMC10045665

[34] Liu M Lu C. Mobile phone addiction and depressive symptoms among Chinese university students: The mediating role of sleep disturbances and the moderating role of gender. Front Public Health. (2022) 10:965135. doi: 10.3389/fpubh.2022.965135 36203678 PMC9531624

[35] Zhang G Yang X Tu X Ding N Lau JT. Prospective relationships between mobile phone dependence and mental health status among Chinese undergraduate students with college adjustment as a mediator. J Affect Disord. (2020) 260:498–505. doi: 10.1016/j.jad.2019.09.047 31539686

[36] Li L Niu Z Mei S Griffiths MD. A network analysis approach to the relationship between fear of missing out (FoMO), smartphone addiction, and social networking site use among a sample of Chinese university students. Comput Hum Behavior. (2022) 128:107086. doi: 10.1016/j.chb.2021.107086

[37] Shoukat S. Cell phone addiction and psychological and physiological health in adolescents. EXCLI J. (2019) 18:47–50. doi: 10.17179/excli2018-2006 30956638 PMC6449671

[38] Sejbuk M Mirończuk-Chodakowska I Witkowska AM. Sleep quality: a narrative review on nutrition, stimulants, and physical activity as important factors. Nutrients. (2022) 14:1912. doi: 10.3390/nu14091912 35565879 PMC9103473

[39] Park SY Choi TS Kim DH Ryu BH Lee SB. Correlation between neck and shoulder pain, neck and shoulder disability, headache and smartphone addiction in adults with sleep disorders. J Korean Soc Phys Med. (2020) 15:43–50. doi: 10.13066/kspm.2020.15.3.43. 박세연.

[40] Billings ME Hale L Johnson DA. Physical and social environment relationship with sleep health and disorders. Chest. (2020) 157:1304–12. doi: 10.1016/j.chest.2019.12.002 PMC7268445 31870910

[41] Krizan Z Boehm NA Strauel CB. How emotions impact sleep: a quantitative review of experiments. Sleep Med Rev. (2024) 74:101890. doi: 10.1016/j.smrv.2023.101890 38154235

[42] Ding X Wang X Yang Z Tang R Tang Y. Relationship between trait mindfulness and sleep quality in college students: a conditional process model. Front Psychol. (2020) 11:576319. doi: 10.3389/fpsyg.2020.576319 33132983 PMC7550415

[43] Zhang L Bai Y Cui X Cao G Li D Yin H. Negative emotions and brain: negative emotions mediates the association between structural and functional variations in emotional-related brain regions and sleep quality. Sleep Med. (2022) 94:8–16. doi: 10.1016/j.sleep.2022.03.023 35447402

[44] Zhang Y Li B Wang J Yang J Zhang L. Effect of mindfulness trait factors on sleep quality of college students: the mediating role of cognitive emotion regulation strategies and anxiety. Chin J Behav Med Brain Sci. (2019), 788–92.

[45] Wieman ST Hall KAA MacDonald HZ Gallagher MW Suvak MK Rando AA. Relationships among sleep disturbance, reward system functioning, anhedonia, and depressive symptoms. Behav Ther. (2022) 53:105–18. doi: 10.1016/j.beth.2021.06.006 35027152

[46] Zhang J Li X Tang Z Xiang S Tang Y Hu W. Effects of stress on sleep quality: multiple mediating effects of rumination and social anxiety. Psicologia: Reflexão e Crítica. (2024) 37:10. doi: 10.1186/s41155-024-00294-2 PMC10948653 38498281

[47] Li T Xie Y Tao S Yang Y Xu H Zou L. Chronotype, sleep, and depressive symptoms among Chinese college students: a cross-sectional study. Front Neurology. (2020) 11:592825. doi: 10.3389/fneur.2020.592825 PMC7773835 33391156

[48] Hu B Wu Q Xie Y Guo L Yin D. Cell phone addiction and sleep disturbance among medical students in Jiangsu Province, China: the mediating role of psychological resilience and the moderating role of gender. Front Psychiatry. (2024) 15:1405139. doi: 10.3389/fpsyt.2024.1405139 38812482 PMC11135470

[49] Lindqvist A Sendén MG Renström EA. What is gender, anyway: a review of the options for operationalising gender. Psychol sexuality. (2021) 12:332–44. doi: 10.1080/19419899.2020.1729844

[50] Pryzgoda J Chrisler JC. Definitions of gender and sex: the subtleties of meaning. Sex roles. (2000) 43:553–69. doi: 10.1023/A:1007123617636

[51] Yue H Zhang X Sun J Liu M Li C Bao H. The relationships between negative emotions and latent classes of smartphone addiction. PloS One. (2021) 16:e0248555. doi: 10.1371/journal.pone.0248555 33720952 PMC7959355

[52] Li L Lok GK Mei SL Cui XL Li L Ng CH. The severity of mobile phone addiction and its relationship with quality of life in Chinese university students. PeerJ. (2020) 8:e8859. doi: 10.7717/peerj.8859 32547849 PMC7271884

[53] Gao W Ping S Liu X. Gender differences in depression, anxiety, and stress among college students: a longitudinal study from China. J Affect Disord. (2020) 263:292–300. doi: 10.1016/j.jad.2019.11.121 31818792

[54] Graves BS Hall ME Dias-Karch C Haischer MH Apter C. Gender differences in perceived stress and coping among college students. PloS One. (2021) 16:e0255634. doi: 10.1371/journal.pone.0255634 34383790 PMC8360537

[55] Franco P Putois B Guyon A Raoux A Papadopoulou M Guignard-Perret A. Sleep during development: sex and gender differences. Sleep Med Rev. (2020) 51:101276. doi: 10.1016/j.smrv.2020.101276 32109833

[56] Goel N Kim H Lao RP. Gender differences in polysomnographic sleep in young healthy sleepers. Chronobiology Int. (2005) 22:905–15. doi: 10.1080/07420520500263235 16298775

[57] Glavin EE Matthew J Spaeth AM. Gender differences in the relationship between exercise, sleep, and mood in young adults. Health Educ Behavior. (2022) 49:128–40. doi: 10.1177/1090198120986782 33576253

[58] Toscano-Hermoso MD Arbinaga F Fernández-Ozcorta EJ Gómez-Salgado J Ruiz-Frutos C. Influence of sleeping patterns in health and academic performance among university students. Int J Environ Res Public Health. (2020) 17:2760. doi: 10.3390/ijerph17082760 32316249 PMC7215924

[59] Leung L. Linking psychological attributes to addiction and improper use of the mobile phone among adolescents in Hong Kong. J Children Media. (2008) 2:93–113. doi: 10.1080/17482790802078565

[60] Xu X. Self-control deficits and their mechanisms in cell phone addicted college students. Southwest University, Chongqing (2014). dissertation/master’s thesis.

[61] Watson D Clark LA Tellegen A. Development and validation of brief measures of positive and negative affect: The PANAS scales. J Pers Soc Psychol. (1988) 54:1063–70. doi: 10.1037/0022-3514.54.6.1063 3397865

[62] Buysse DJ Reynolds CF III Monk TH Berman SR Kupfer DJ. The Pittsburgh Sleep Quality Index: A new instrument for psychiatric practice and research. Psychiatry Res. (1989) 28:193–213. doi: 10.1016/0165-1781(89)90047-4 2748771

[63] Fritz MS MacKinon DP. Required sample size to detect the mediated effect. psychol Science. (2007) 18:233–9. doi: 10.1111/j.1467-9280.2007.01882.x PMC2843527 17444920

[64] Igartua JJ Hayes AF. Mediation, moderation, and conditional process analysis: Concepts, computations, and some common confusions. Spanish J Psychol. (2021) 24:e49. doi: 10.1017/SJP.2021.46 35923144

[65] Liu Q Zhou Z Niu G Fan C. Mobile phone addiction and sleep quality in adolescents: Mediation and moderation analyses. Acta Psychologica Sinica. (2017) 49:1524–36. doi: 10.3724/SP.J.1041.2017.01524

[66] Demirci K Akgönül M Akpinar A. Relationship of smartphone use severity with sleep quality, depression, and anxiety in university students. J Behav Addictions. (2015) 4:85–92. doi: 10.1556/2006.4.2015.010 PMC4500888 26132913

[67] Woods HC Scott H. Sleepyteens: Social media use in adolescence is associated with poor sleep quality, anxiety, depression and low self-esteem. J Adolescence. (2016) 51:41–9. doi: 10.1016/j.adolescence.2016.05.008 27294324

[68] Sahin S Ozdemir K Unsal A Temiz N. Evaluation of mobile phone addiction level and sleep quality in university students. Pakistan J Med Sci. (2013) 29:913–8. doi: 10.12669/pjms.294.3686 PMC3817775 24353658

[69] Lemola S Perkinson-Gloor N Brand S Dewald-Kaufmann JF Grob A. Adolescents’ electronic media use at night, sleep disturbance, and depressive symptoms in the smartphone age. J Youth Adolescence. (2015) 44:405–18. doi: 10.1007/s10964-014-0176-x 25204836

[70] Lepp A Barkley JE Sanders GJ Rebold M Gates P. The relationship between cell phone use, physical and sedentary activity, and cardiorespiratory fitness in a sample of US college students. Int J Behav Nutr Phys Activity. (2013) 10:1–9. doi: 10.1186/1479-5868-10-79 PMC3693866 23800133

[71] Riemann D Spiegelhalder K Feige B Voderholzer U Berger M Perlis M. The hyperarousal model of insomnia: A review of the concept and its evidence. Sleep Med Rev. (2010) 14:19–31. doi: 10.1016/j.smrv.2009.04.002 19481481

[72] Thomée S. Mobile phone use and mental health. A review of the research that takes a psychological perspective on exposure. Int J Environ Res Public Health. (2018) 15:2692. doi: 10.3390/ijerph15122692 30501032 PMC6314044

[73] Loughran SP Wood AW Barton JM Croft RJ Thompson B Stough C. The effect of electromagnetic fields emitted by mobile phones on human sleep. Neuroreport. (2005) 16:1973–6. doi: 10.1097/01.wnr.0000186593.79705.3c 16272890

[74] Chang AM Aeschbach D Duffy JF Czeisler CA. Evening use of light-emitting eReaders negatively affects sleep, circadian timing, and next-morning alertness. Proc Natl Acad Sci. (2015) 112:1232–7. doi: 10.1073/pnas.1418490112 PMC4313820 25535358

[75] Chen L Yan Z Tang W Yang F Xie X He J. Mobile phone addiction levels and negative emotions among Chinese young adults: The mediating role of interpersonal problems. Comput Hum Behavior. (2016) 55:856–66. doi: 10.1016/j.chb.2015.10.030

[76] Çağan Ö Ünsal A Çelik N. Evaluation of college students’ the level of addiction to cellular phone and investigation on the relationship between the addiction and the level of depression. Procedia-Social Behav Sci. (2014) 114:831–9. doi: 10.1016/j.sbspro.2013.12.793

[77] Kales A Caldwell AB Preston TA Healey S Kales JD. Personality patterns in insomnia: Theoretical implications. Arch Gen Psychiatry. (1976) 33:1128–34. doi: 10.1001/archpsyc.1976.01770090118013 962495

[78] Jansson-Fröjmark M Lindblom K. A bidirectional relationship between anxiety and depression, and insomnia? A prospective study in the general population. J Psychosomatic Res. (2008) 64:443–9. doi: 10.1016/j.jpsychores.2007.10.016 18374745

[79] Staner L. Sleep and anxiety disorders. Dialogues Clin Neurosci. (2003) 5:249–58. doi: 10.31887/DCNS.2003.5.3/lstaner PMC3181635 22033804

[80] Vandekerckhove M Weiss R Schotte C Exadaktylos V Haex B Verbraecken J. The role of presleep negative emotion in sleep physiology. Psychophysiology. (2011) 48:1738–44. doi: 10.1111/j.1469-8986.2011.01281.x 21895689

[81] Hobfoll SE Freedy J Lane C Geller P. Conservation of social resources: Social support resource theory. J Soc Pers Relationships. (1990) 7:465–78. doi: 10.1177/0265407590074004

[82] Garnefski N Kraaij V Spinhoven P. Negative life events, cognitive emotion regulation and emotional problems. Pers Individ Differences. (2001) 30:1311–27. doi: 10.1016/S0191-8869(00)00113-6

[83] Liang L Zhu M Dai J Li M Zheng Y. The mediating roles of emotional regulation on negative emotion and internet addiction among Chinese adolescents from a development perspective. Front Psychiatry. (2021) 12:608317. doi: 10.3389/fpsyt.2021.608317 33897485 PMC8062778

[84] Silvers JA. Adolescence as a pivotal period for emotion regulation development. Curr Opin Psychol. (2022) 44:258–63. doi: 10.1016/j.copsyc.2021.09.023 34781238

[85] Talley G Shelley-Tremblay J. The relationship between mindfulness and sleep quality is mediated by emotion regulation. Psychiatry Int. (2020) 1:42–66. doi: 10.3390/psychiatryint1020007

[86] Zhang M Ding Y Zhang J Jiang X Xu N Zhang L. Effect of group impromptu music therapy on emotional regulation and depressive symptoms of college students: a randomized controlled study. Front Psychol. (2022) 13:851526. doi: 10.3389/fpsyg.2022.851526 35432107 PMC9008882

[87] Wang Y Tian J Yang Q. Tai Chi exercise improves working memory capacity and emotion regulation ability. Front Psychol. (2023) 14:1047544. doi: 10.3389/fpsyg.2023.1047544 36874821 PMC9983368

[88] Thomsen DK Mehlsen MY Viidik A Sommerlund B Zachariae R. Age and gender differences in negative affect—is there a role for emotion regulation? Pers Individ Differences. (2005) 38:1935–46. doi: 10.1016/j.paid.2004.12.001

[89] Nolen-Hoeksema S Larson J Grayson C. Explaining the gender difference in depressive symptoms. J Pers Soc Psychol. (1999) 77:1061–72. doi: 10.1037/0022-3514.77.5.1061 10573880

[90] McRae K Ochsner KN Mauss IB Gabrieli JJD Gross JJ. Gender differences in emotion regulation: an fMRI study of cognitive reappraisal. Group Processes Intergroup Relations. (2008) 11:143–62. doi: 10.1177/1368430207088035 PMC5937254 29743808

[91] Masumoto K Taishi N Shiozaki M. Age and gender differences in relationships among emotion regulation, mood, and mental health. Gerontology Geriatric Med. (2016) 2:2333721416637022. doi: 10.1177/2333721416637022 PMC5119800 28138490

[92] Mauss IB Troy AS LeBourgeois MK. Poorer sleep quality is associated with lower emotion-regulation ability in a laboratory paradigm. Cogn Emotion. (2013) 27:567–76. doi: 10.1080/02699931.2012.727783 PMC3931554 23025547

[93] Smith R Persich MR Chuning AE Cloonan S Woods-Lubert R Skalamera J. Improvements in mindfulness, interoceptive and emotional awareness, emotion regulation, and interpersonal emotion management following completion of an online emotional skills training program. Emotion. (2024) 24:431–50. doi: 10.1037/emo0001237 PMC10837318 37535567

[94] Yuan J Luo Y Yan JH Meng X Yu F Li H. Neural correlates of the females’ susceptibility to negative emotions: An insight into gender-related prevalence of affective disturbances. Hum Brain Mapping. (2009) 30:3676–86. doi: 10.1002/hbm.20796 PMC6870863 19404991

